# Treatment Heterogeneity in *Pseudomonas aeruginosa* Pneumonia

**DOI:** 10.3390/antibiotics11081033

**Published:** 2022-07-30

**Authors:** Aisling R. Caffrey, Haley J. Appaneal, J. Xin Liao, Emily C. Piehl, Vrishali Lopes, Laura A. Puzniak

**Affiliations:** 1 Infectious Diseases Research Program, Providence Veterans Affairs Medical Center, Providence, RI 02908, USA; haley.appaneal@gmail.com (H.J.A.); jxliao@uri.edu (J.X.L.); ec.piehl@gmail.com (E.C.P.); vrishali.lopes@va.gov (V.L.); 2 Center of Innovation in Long-Term Support Services, Providence Veterans Affairs Medical Center, Providence, RI 02908, USA; 3 College of Pharmacy, University of Rhode Island, Kingston, RI 02881, USA; 4 School of Public Health, Brown University, Providence, RI 02903, USA; 5 Merck & Co., Inc., Rahway, NJ 07065, USA; laurapuzniak@yahoo.com

**Keywords:** *Pseudomonas aeruginosa*, pneumonia, treatment heterogeneity, antibiotics, anti-pseudomonal antibiotics

## Abstract

We have previously identified substantial antibiotic treatment heterogeneity, even among organism-specific and site-specific infections with treatment guidelines. Therefore, we sought to quantify the extent of treatment heterogeneity among patients hospitalized with *P. aeruginosa* pneumonia in the national Veterans Affairs Healthcare System from Jan-2015 to Apr-2018. Daily antibiotic exposures were mapped from three days prior to culture collection until discharge. Heterogeneity was defined as unique patterns of antibiotic treatment (drug and duration) not shared by any other patient. Our study included 5300 patients, of whom 87.5% had unique patterns of antibiotic drug and duration. Among patients receiving any initial antibiotic/s with a change to at least one anti-pseudomonal antibiotic (n = 3530, 66.6%) heterogeneity was 97.2%, while heterogeneity was 91.5% in those changing from any initial antibiotic/s to only anti-pseudomonal antibiotics (n = 576, 10.9%). When assessing heterogeneity of anti-pseudomonal antibiotic classes, irrespective of other antibiotic/s received (n = 4542, 85.7%), 50.5% had unique patterns of antibiotic class and duration, with median time to first change of three days, and a median of two changes. Real-world evidence is needed to inform the development of treatment pathways and antibiotic stewardship initiatives based on clinical outcome data, which is currently lacking in the presence of such treatment heterogeneity.

## 1. Introduction

*Pseudomonas aeruginosa* is an important nosocomial pathogen, often causing pneumonia in hospitalized patients [1,2]. Second only to *Staphylococcus aureus*, *P. aeruginosa* is the most common Gram-negative pathogen isolated from hospitalized patients with bacterial pneumonia [3]. *P. aeruginosa* is the fourth most common cause of nosocomial infections, and the leading Gram-negative cause of both hospital-acquired (HABP) and ventilator-acquired bacterial pneumonia (VABP) in the United States (US) and an important nosocomial pathogen worldwide [3,4,5,6,7]. Further, *P. aeruginosa* is significant respiratory tract pathogen in community-acquired pneumonia (CAP) among patients with certain comorbidities and/or history of *P. aeruginosa* colonization or infection [3,8,9].

The greatest challenges with *P. aeruginosa* infections include its virulence and its intrinsic, acquired, and adaptive mechanisms of antibiotic resistance, often expressed simultaneously; which, have made infections caused by this pathogen particularly difficult to treat [4,10]. The frequency of multi-drug resistant (MDR) strains is increasing, and MDR *P. aeruginosa* has been recognized by the Centers for Disease Control and Prevention (CDC) as a serious public health threat [11,12,13]. Between 17% and 39% of *P. aeruginosa* VABP isolates are MDR in the US [4]. MDR *P. aeruginosa* nosocomial pneumonia is associated with increased morbidity, mortality, length of hospital and intensive care unit stay, and increased health care costs, likely related to inappropriate empiric therapy [14,15,16,17,18,19,20].

Existing treatment guidelines for *P. aeruginosa* include the 2019 Infectious Diseases Society of America (IDSA) and American Thoracic Society (ATS) CAP guidelines and newer treatment guidelines from IDSA for resistant Gram-negative infections [3,8,21,22]. However, treatment of *P. aeruginosa* pneumonia can vary based on patient-specific factors, such as antibiotic allergies, drug toxicities and interactions, comorbidities, and the substantial heterogeneity of possible resistance mechanisms at play with *P. aeruginosa*. While there is frequent discussion surrounding heterogeneity of patients and heterogeneity of treatment effects, there is very little work evaluating differences in how patients are treated (treatment heterogeneity). We do not have a good understanding of how patients are treated beyond a single hospital or clinic. Furthermore, definitions of treatment in research tend to be overly simplistic and broad, leading to substantial misclassification of treatment definitions. Therefore, real-world evidence must be interpreted with caution. Over the past several years, our group has been studying treatment heterogeneity in serious bacterial infections, defined as drug-resistant infections requiring hospitalization and associated with high mortality such as pneumonia and/or bloodstream infections [23]. We have found that even in the presence of clinical guidelines, society guidelines, and/or hospital protocols to guide treatment for the optimal management of serious bacterial infections, substantial heterogeneity exists. We therefore wanted to assess whether heterogeneity persists in infections where there is limited evidence to support treatment guidelines, specifically *P. aeruginosa* pneumonia.

## 2. Results

### 2.1. Study Population

Our study included 5300 hospitalized patients from 112 hospitals in the national Veterans Affairs (VA) Healthcare system, with a *P. aeruginosa* isolated from respiratory tract cultures collected between January 2015 and April 2018. Our study population was mostly male (n = 5167, 97.5%), older (mean age 70.4 years, standard deviation [SD] 10.3; Table 1), and White (n = 3956, 74.6%). About one-third (n = 1800, 34.0%) of patients received care in intensive care units (ICU), and the comorbidity burden was high (median Charlson 3, interquartile range [IQR] 2–5; median Elixhauser 5, IQR 3–7]). Multidrug-resistant *P. aeruginosa* respiratory isolates were present in 12.4% (n = 636/5117) patients, with 22.0% (n = 1136/5157) demonstrating fluoroquinolone resistance and 19.0% (n = 874/4601) demonstrating carbapenem resistance. The median time from admission to *P. aeruginosa* culture was 2 days (IQR 0–7).

### 2.2. Treatment Heterogeneity

**Figure 1:**
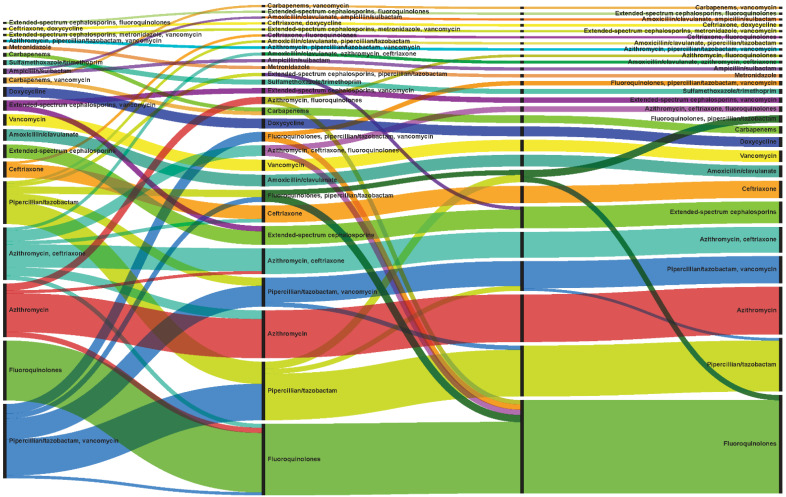
Common treatment patterns. Alluvial chart demonstrates the 50 most common unique antibiotic treatment patterns (patterns alone without duration of therapy) observed among all patients (n = 5300), including changes in treatment, from three days prior to the culture collection date until discharge, or 30 days from culture for longer hospital stays. The width of each line represents the number of patients receiving that specific treatment. Patients without changes in treatment are depicted in each segment as having the same antibiotic as the previous segment, while those with changes, move to another antibiotic in the next segment. Different colors represent different antibiotics or concomitant antibiotics.

When evaluating all antibiotics, 87.5% (n = 4635) of patients with *P. aeruginosa* pneumonia had unique antibiotic treatments, in terms of the specific antibiotic, timing of receipt of each antibiotic (which day of admission), and duration of therapy (Table 2). Most (n = 4473, 84.4%) patients had a change in therapy, with a median of three changes over the course of the hospitalization. Most changes (43.5%) were made the day after culture collection, with a median time to first change of 1 day (IQR 0–2), median time to second change of 2 days (IQR 1–4), and median time to third change of 4 days (2–6). Figure 1 demonstrates the 50 most common unique antibiotic treatment patterns (patterns alone without duration of therapy), including changes in therapy, over the course of the admission [24].

Among patients with any initial antibiotic therapy (monotherapy or combination therapy), and changing to at least one anti-pseudomonal antibiotic (n = 3530, 66.6%), heterogeneity was higher at 97.2% (n = 3430/3530), with a median of three changes. Among patients initially treated with any antibiotic and then changing to only anti-pseudomonal antibiotics (n = 576, 10.9%), heterogeneity was 91.5% (n = 527/576). When assessing anti-pseudomonal antibiotic classes only, irrespective of other antibiotics received (n = 4542, 85.7%), heterogeneity was 50.5% (n = 2294/4542) and among those with changes in therapy, there were a median of 2 changes, with the first change most commonly occurring 3 days after culture.

**Figure 2:**
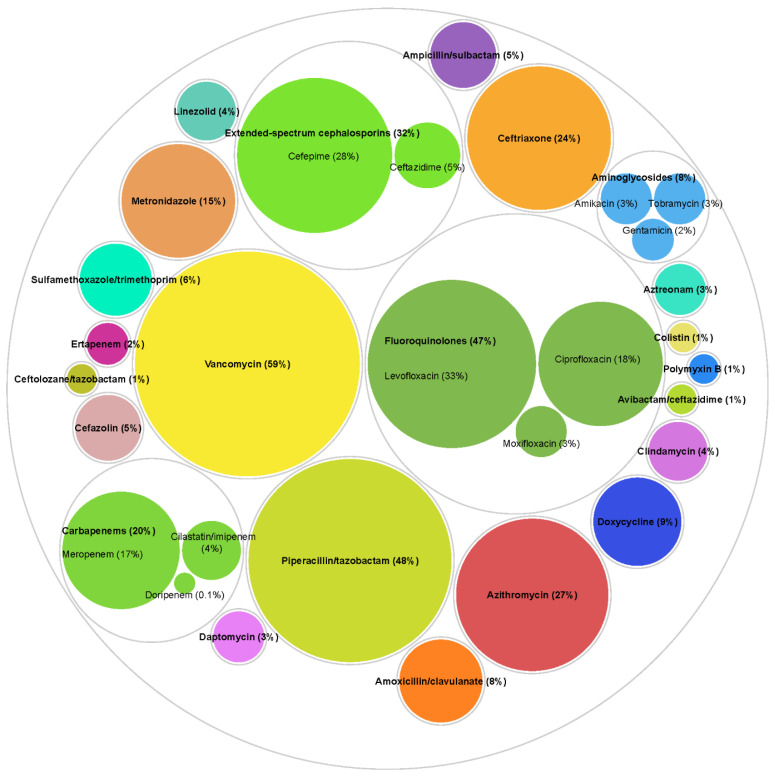
Utilization of specific antibiotics. Antibiotic exposures were assessed from three days prior to Pseudomonas aeruginosa culture collection date until discharge, or 30 days from culture for longer hospital stays among all patients (n = 5300). Counts and percentages are not mutually exclusive as 88.9% patients received more than one antibiotic over the course of the admission (median number of antibiotics received 3, interquartile range 2–5). Different colors represent different antibiotics. Other antibiotics not included in the figure were used in <2% of patients.

The most commonly utilized antibiotics (Figure 2) [24] were vancomycin (n = 3106, 58.6%), piperacillin/tazobactam (n = 2563, 48.4%), levofloxacin (n = 1766, 33.3%), cefepime (n = 1475, 27.8%), azithromycin (n = 1447, 27.3%), ceftriaxone (n = 1259, 23.8%), ciprofloxacin (n = 931, 17.6%), meropenem (n = 876, 16.5%), and metronidazole (n = 809, 15.3%). Most patients (88.9%) were treated more than one antibiotic over the course of the admission (median antibiotics received 3, IQR 2–5). The utilization distribution of anti-pseudomonal antibiotic classes was 47.4% (n = 2510) for fluoroquinolones, 31.9% (n = 1692) for extended-spectrum cephalosporins, 19.7% (n = 1045) for carbapenems, 8.3% (n = 440) for aminoglycosides.

When assessing anti-pseudomonal antibiotic classes only (n = 4542, 85.7%), irrespective of other antibiotics received and without considering duration of each antibiotic, only eight patterns were observed in more than 2% of patients: (1) piperacillin/tazobactam (15.8%), (2) fluoroquinolone (13.3%), (3) extended-spectrum cephalosporin (8.0%), (4) initial treatment with piperacillin/tazobactam alone, then a change to combination therapy of a fluoroquinolone and piperacillin/tazobactam, and then a change to a fluoroquinolone alone (discontinuation of piperacillin/tazobactam) (4.8%), (5) initial treatment with piperacillin/tazobactam alone, then a change to combination therapy of a fluoroquinolone and piperacillin/tazobactam (2.9%), (6) initial treatment with an extended-spectrum cephalosporin, then a change to combination therapy of a fluoroquinolone and an extended-spectrum cephalosporin, and then a change to a fluoroquinolone alone (discontinuation of extended-spectrum cephalosporin) (2.4%), (7) carbapenem (2.3%), and (8) initial treatment with piperacillin/tazobactam, then a change to combination therapy of an extended-spectrum cephalosporin and piperacillin/tazobactam, and then a change to an extended-spectrum cephalosporin alone (discontinuation of piperacillin/tazobactam) (2.1%).

### 2.3. Clinical Outcomes

Most patients had changes in therapy (n = 4473, 84.4%). Length of hospital stay, mortality, and persistent positive cultures were significantly higher among those who had changes in therapy (Table 3, median length of stay admission to discharge 12 versus 4 days, inpatient mortality 17.1% versus 10.3%, persistent positive culture 38.1% versus 9.2%) as compared to those without changes in therapy. Among those without changes in therapy (n = 827), length of stay and mortality were significantly lower among those treated with monotherapy (n = 591, 71.5%) (median length of stay admission to discharge 5 versus 3 days, inpatient mortality 21.2% versus 5.9%) as compared to those treated with combination therapy.

## 3. Discussion

Among hospitalized patients with positive *P. aeruginosa* respiratory cultures, 87.5% had different antibiotic treatment patterns, in terms of all antibiotics received each day and the duration of each antibiotic therapy. This heterogeneity was largely driven by differences in antibiotic treatment patterns as opposed to duration of therapy (75.5% heterogeneity when not considering length of therapy versus 87.5% heterogeneity when including length of therapy). Treatment heterogeneity remained high, at approximately 50%, even when restricting the analysis to anti-pseudomonal antibiotic classes only (not accounting for other antibiotics received) and duration of therapy, with duration accounting for approximately half of the heterogeneity observed (heterogeneity of 23.1% when not considering length of therapy). Most changes were made the day after culture collection, which was likely when preliminary culture results were communicated to the treating clinicians. Current methods to assess treatment, including intent-to-treat, as-treated, and even time-dependent exposures, do not adequately account for the extensive heterogeneity observed in the treatment of infectious diseases. This misclassification has important implications as clinical outcomes may vary between heterogeneous treatment approaches, which makes it difficult to attribute outcomes to one specific treatment approach. Future work should investigate the impact of specific treatment approaches on clinical outcomes in patients with *P. aeruginosa* pneumonia.

Inpatient mortality was highest among those initially receiving combination therapy and continuing with that same combination therapy (no changes in therapy), and lowest among those receiving only monotherapy (no changes in therapy). While this could indicate that initial targeted anti-pseudomonal monotherapy (monotherapy not requiring changes in therapy) may be associated with better clinical outcomes, these patients may have also been less complex, with less severe infections. It is also possible that since patients exposed to combination therapy were exposed to more antibiotics, they had a higher risk for toxicity and adverse drug events than those exposed to monotherapy. As such, these differences in outcomes require further study.

We observed higher rates of mortality in our study compared to the 8% mortality rate reported by the CDC in 2019 for MDR *P. aeruginosa* infections, which included both respiratory and non-respiratory infections [13]. As worse outcomes, including mortality, have been associated with MDR *P. aeruginosa* infections, and only 12.4% of our population had MDR *P. aeruginosa*, the clinical significance of pneumonias caused by all *P. aeruginosa*, including both MDR and susceptible strains, cannot be understated [25,26,27,28,29].

Empiric treatment recommendations for *P. aeruginosa* pneumonia endorsed by the IDSA include piperacillin-tazobactam (4.5 g every 6 h), cefepime (2 g every 8 h), ceftazidime (2 g every 8 h), imipenem (500 mg every 6 h), meropenem (1 g every 8 h), or aztreonam (2 g every 8 h) [3,8]. Newer treatment guidelines from IDSA for resistant Gram-negative infections recommend monotherapy with ceftolozane-tazobactam, ceftazidime-avibactam, or imipenem-cilastatin-relebactam for infections outside the urinary tract caused by difficult to treat (DTR) *P. aeruginosa* (defined as non-susceptibility to all of the following: piperacillin-tazobactam, ceftazidime, cefepime, aztreonam, meropenem, imipenem-cilastatin, ciprofloxacin, and levofloxacin) [21,22]. If none of these newer beta-lactam/beta-lactamase inhibitors are options due to intolerance or resistance, alternative treatment options include cefiderocol or an aminoglycoside, such as plazomicin [21]. However, it should be noted there is limited clinical data supporting treatment recommendations of DTR *P. aeruginosa* and older treatment options, such as aminoglycosides and colistin, are recognized to have significant toxicity issues [21].

While existing guidelines address empiric treatment options for *P. aeruginosa* pneumonias, given the complexity and variability of antibiotic resistance patterns among *P. aeruginosa* strains causing infection, guidelines may benefit from specific and tailored recommendations. For example, the use of guideline-recommended empiric therapy for highly resistant *P. aeruginosa* strains could lead to initial treatment failure, while empiric therapy may be excessively broad for more sensitive *P. aeruginosa* strains, which could lead to longer hospital stays, and increased risk of developing additional resistance or *Clostridioides difficile* infections [30,31,32,33].

The fundamental principle in treatment of serious *P. aeruginosa* infections is early administration of appropriate antibiotics [34]. Previous work has demonstrated that the odds of mortality in patients with *P. aeruginosa* pneumonia is up to 3 times higher in those treated with inappropriate versus appropriate empiric treatment [35,36]. As such, best practice guidelines for community-acquired and HABP/VABP both recommend empiric treatment with conventional anti-pseudomonal β-lactams (meropenem, imipenem, doripenem, piperacillin-tazobactam, cefepime, and ceftazidime), potentially in combination with a second agent (aminoglycoside, fluoroquinolone, or polymyxin), when *P. aeruginosa* is suspected [8,34,37]. Two agents are generally recommended until bacterial susceptibly results are available, if the patient has specific risk factors for MDR *P. aeruginosa*, such as high risk for mortality, previous intravenous anti-pseudomonal antibiotic therapy, and/or based on local susceptibility data [8,34,37,38,39]. These recommendations are centered around the goals of achieving early appropriate therapy while also limiting superfluous coverage, which increases the risk of adverse drug effects, *Clostridioides difficile* infections, and antimicrobial resistance [37].

Use of newer β-lactam antibiotics, such as ceftolozane/tazobactam, ceftazidime/avibactam, cefiderocol, and imipenem-cilastatin-relebactam, may also be considered for treatment of *P. aeruginosa* pneumonias. Empiric or targeted use of newer agents may increase earlier receipt of appropriate therapy, particularly for resistant *P. aeruginosa* infections, while decreasing the risk of unintended effects from combination therapy with older, more toxic antibiotics [40,41]. However, their role in therapy has yet to be fully elucidated and these agents remain largely absent from guidelines [34]. For directed therapy, while it has been postulated that continued combination therapy may minimize the emergence of resistance and promote antimicrobial synergy, there is a lack of data to support these hypotheses [42,43]. As such, once culture results are available, it is generally recommended to streamline treatment to a single, highly active agent for the directed treatment of *P. aeruginosa* pneumonia [34]. Despite these general recommendations, there is a lack of high-level evidence to firmly guide treatment decisions, especially for patients with infections due to resistant phenotypes, in part, because it is not a requirement for the approval of new antibiotics [44].

Treatment patterns observed in our study were consistent with best practice guidelines for the treatment of CAP and HABP/VABP guidelines [3,8,9,21,37]. It is generally recommended that a β-lactam plus a macrolide or a respiratory fluroquinolone are used for empiric treatment of CAP in hospitalized patients without suspicion for *P. aeruginosa*, which is consistent with our findings regarding the frequency of use of ceftriaxone (23.8%), azithromycin (27.3%), and levofloxacin (33.3%). For HABP/VABP, piperacillin/tazobactam or cefepime are generally recommended for empiric treatment, with the addition of vancomycin if there are risk factors for methicillin-resistant *Staphylococcus aureus* (MRSA) or the local prevalence of MRSA is high (>20%) [8]. While *P. aeruginosa* is intrinsically resistant to ceftriaxone, azithromycin, and vancomycin, these represent recommended empiric treatment options for CAP (ceftriaxone and azithromycin) [8] and MRSA (vancomycin) [37], and do not preclude their use for the treatment of concomitant infections or syndromes, such as chronic obstructive pulmonary disease or another infection type. For directed anti-pseudomonal therapy, single-agent conventional therapy (piperacillin-tazobactam, cefepime, and ceftazidime) based on susceptibilities is generally recommended, with carbapenems (meropenem, imipenem, doripenem) potentially being reserved for more resistant phenotypes or polymicrobial infections [8,37].

Real-world data evaluating *P. aeruginosa* pneumonia treatments are limited. A recent, observational cohort study compared ceftolozane/tazobactam to polymyxin or aminoglycoside-based regimens for the treatment of resistant *P. aeruginosa* infections (52% VABP) [45]. This study similarly demonstrated the frequent use of conventional anti-pseudomonal therapy in the polymyxin or aminoglycoside-based combination treatment group with carbapenems, piperacillin/tazobactam, and extended-spectrum cephalosporins most commonly being used. However, detailed descriptions of treatment patterns were not described, nor were additional agents used for any polymicrobial infections or concomitant infections.

Resistance rates in our cohort were lower than previous reports, with 12.4% demonstrating MDR, 13.1% with aminoglycoside resistance, 19.0% with carbapenem resistance, 17.2% with extended-spectrum cephalosporin resistance, 22.0% with fluoroquinolone resistance, and 15.3% with piperacillin resistance. Alternatively, of 1412 *P. aeruginosa* isolates from adult patients with VAP submitted to the National Healthcare Safety Network (NHSN) between 2015–2017, between 17 and 39% were MDR, dependent on hospital location [4]. In the NHSN, reported rates of resistance in VABP *P. aeruginosa* were generally higher than rates observed in our study (aminoglycosides 13.6–29.9%, carbapenems 26.3–61.4%, extended-spectrum cephalosporin 25.9–44.7%, fluoroquinolones 26.7–45.8%, and piperacillin/tazobactam 21.7–34.8%) [4]. Rates of resistance may have been lower if the NHSN data included isolates from CAP. Similarly, of 258 patients with *P. aeruginosa* nosocomial pneumonia from 3 hospitals in the US, 20.5% were MDR, and reported resistance rates for each class of antibiotics were higher than those we observed (aminoglycosides 19.2%, carbapenems 21%, extended-spectrum cephalosporin 18.6%, fluoroquinolones 24.1%, and piperacillin/tazobactam 17.4%) [14]. Twenty-years of surveillance data (1997–2016) from the worldwide SENTRY Antimicrobial Surveillance Program also demonstrated higher rates of resistance than we observed in our cohort [46]. The overall rate of MDR clinical *P. aeruginosa* isolates was 24.9%, with the highest rates among those with pneumonia (27.7%) [46]. Isolates with MDR phenotypes were less common in North America (18.9%) than other regions worldwide, but still higher than the rate we observed.

Despite general recommendations, there are limited comparative data to support the most optimal agent/s to use for treatment of *P. aeruginosa* pneumonia, especially those due to resistant phenotypes. More comparative clinical data is needed to identify if there are preferred antibiotic agents overall for *P. aeruginosa* pneumonia or if there are clinical scenarios in which certain agents may be preferred [34,42]. Additionally, more data is needed to identify when combination therapy may be most beneficial to patients with *P. aeruginosa* pneumonia and what antibiotic combinations are associated with the highest effectiveness and safety. Additional data may also be important to determine if there is a need to tailor antibiotic regimens based on the risk or presence of MDR isolates. These gaps in knowledge need to be filled with strong real-world clinical data for guidelines and hospital protocols to outline optimal therapy for patients with serious *P. aeruginosa* infections. The high treatment heterogeneity we observed, even despite clinicians commonly using recommended agents, highlights the continued uncertainty surrounding optimal treatment of *P. aeruginosa* infections and the need for clearer, more detailed evidence-based specific treatment guidelines based on patient risk factors and/or local epidemiology.

There are limitations to this retrospective observational study. We were unable to distinguish between true infection versus colonization as clinical signs and symptoms and signs of pneumonia were not captured. However, 72% of patients had a pneumonia diagnosis during the admission. It is possible that the treatment captured may have been for another infection or for a concomitant and/or polymicrobial infection. Furthermore, we were also unable to classify the type of pneumonia (HABP/VABP/vHABP versus CAP) diagnosed. Given the differential burden of pseudomonal HABP/VABP/vHABP and CAP on mortality as identified in previous studies [17], stratification of these diagnoses could help inform the clinical outcomes seen in our cohort. The reasons for changes in antibiotic therapy are unknown. We measured the time from culture collection to antibiotic change as opposed to time from culture results, including pathogen identification and antimicrobial susceptibilities. Potential reasons for change include possible antibiotic de-escalation or escalation based on pathogen and/or susceptibly results, clinical improvement or worsening of clinical signs and symptoms, or care transitions (such a preparatory to discharge, discontinue intravenous antibiotics for oral).

Our estimate of heterogeneity is likely an underestimate, as it did not include changes in route (for example, intravenous to oral changes) or dose, antibiotics received in other non-VA settings (non-VA nursing home or non-VA hospital), inhaled antibiotics, or post-discharge antibiotics. Similarly, outcomes were only captured among those who were treated in VA facilities and not those who sought treatment outside the VA. Susceptibility testing across the VA Healthcare system is not uniform which may lead to inconsistencies in determining antibiotic resistance and MDR. However, accepted definitions of the CDC NHSN were used to identify MDR phenotypes [47]. While several demographics and clinical characteristics of the study cohort were described, previous antibiotic exposures, one of the main risk factors for resistant pseudomonal infections, were not assessed. The generalizability of this study is limited to patients admitted to VA hospitals. Heterogeneity among VA hospitals may be lower than other non-VA hospitals, as the VA is the nation’s largest integrated healthcare system and while there are no national VA guidelines for treatment of *P. aeruginosa* pneumonia, resources and education are often shared among local VA facilities through national antibiotic stewardship resources and initiatives, such as the national VA Antibiotic Stewardship Task Force.

## 4. Materials and Methods

### 4.1. Study Population

Our study included hospitalized patients ≥18 years old with *P. aeruginosa* isolated from respiratory tract cultures (sputum and/or bronchoalveolar lavage) collected between January 2015 and April 2018 in the national VA Healthcare System. The culture could be collected anytime during admission and only the first hospitalization identified during the study period was selected for inclusion. This study was approved by the Institutional Review Board and Research and Development Committee of the VA Providence Healthcare System.

### 4.2. Exposure Mapping

We utilized exposure mapping to identify all antibiotics received on each day, from three days prior to the culture collection date until discharge, or 30 days from culture for longer hospital stays. With exposure mapping, all antibiotic exposures are captured, which allows assessment of combination therapy, duration of therapy, and changes in therapy. Antibiotic exposures were captured from barcode medication administration records and pharmacy dispensings, which include antibiotics given in the emergency department.

### 4.3. Treatment Heterogeneity

Measures of heterogeneity included number of changes in therapy and time to first change for each patient. Time to first change was assessed to capture initial change from empiric to targeted therapy after initial culture results are communicated to the treating clinician. Treatment patterns were built from daily antibiotic exposures and duration of each antibiotic exposure, and therefore captures all changes in antibiotic treatments day to day. Dose changes and changes from intravenous to oral forms of the same antibiotic were not considered changes in therapy. Exposure mapping was carried out for (1) all antibiotics, (2) any initial antibiotic/s and then at least one anti-pseudomonal antibiotic at the first change in therapy (aminoglycosides: amikacin, gentamicin, tobramycin; carbapenems: imipenem, meropenem, doripenem; extended-spectrum cephalosporins: cefepime, ceftazidime; fluoroquinolones: ciprofloxacin, levofloxacin; piperacillin/tazobactam; aztreonam; polymyxins: colistin, polymyxin B; ceftazidime/avibactam; ceftolozane/tazobactam), (3) any initial antibiotic/s and then only anti-pseudomonal antibiotics after first change, and (4) only assessing anti-pseudomonal antibiotic classes.

The definition of a common, or shared, treatment pattern was at least two patients hospitalized with positive *P. aeruginosa* respiratory tract cultures and treated with the same antibiotic exposures for the same duration. Therefore, unique treatment patterns were those where only a single patient had a specific treatment pattern, assessed either at the antibiotic or as antibiotic class level and accounting for duration. Non-unique treatment patterns were defined as those where multiple admissions had the same pattern of antibiotic exposures and durations. For example, the following was a treatment pattern shared by several patients: piperacillin/tazobactam plus vancomycin for three days, which was changed to only piperacillin/tazobactam for five days. In contrast, this was an example of a unique treatment pattern which was only identified in one patient: amoxicillin/clavulanate for one day, which was changed to piperacillin/tazobactam for six days and then changed to levofloxacin and piperacillin/tazobactam for one day.

### 4.4. Clinical Outcomes

Outcomes assessed included length of stay (from admission and culture collection to discharge), mortality (inpatient and 30-day from culture), readmission within 30 days of discharge, persistent positive *P. aeruginosa* culture, and *P. aeruginosa* reinfection within 30 days of discharge. Persistent positive *P. aeruginosa* culture was defined as a positive sputum and/or bronchoalveolar lavage for *P. aeruginosa* after 7 days of treatment. *P. aeruginosa* reinfection was defined as a positive sputum and/or bronchoalveolar lavage culture for *P. aeruginosa* after discharge.

### 4.5. Statistical Analysis

Outcomes were compared between patients who had changes in therapy and no changes in therapy, and among those who had no changes in therapy, between those treated with monotherapy and combination therapy. Proportions of categorical variables were compared between groups using Chi-square tests (one comparison with expected cell count <5 utilized Fisher’s exact test, as indicted in Table 3) and the non-parametric Wilcoxon test when comparing medians of continuous variables. Statistical significance was considered at a *p*-value < 0.05. Analyses were completed with SAS (Version 9.2; SAS Institute, Inc.: Cary, NC, USA).

## 5. Conclusions

In our retrospective cohort study of 5300 MDR *P. aeruginosa* infections, treatment heterogeneity was high, ranging from 97.2% in patients receiving any initial antibiotic/s with a change to at least one anti-pseudomonal antibiotic, to 50.5% when assessing anti-pseudomonal antibiotic classes, irrespective of other antibiotic received. Real-world evidence is needed to help support and inform the development of treatment protocols and antibiotic stewardship programs based on clinical and patient outcome data, which is currently lacking in the presence of such treatment heterogeneity.

## Figures and Tables

**Table 1:** Demographics and comorbidities among patients with *Pseudomonas aeruginosa* pneumonia.

Demographics		Total N = 5300
Age (years), mean (SD)		70.4 (10.3)
Sex		
	Male	5167 (97.5%)
	Female	133 (2.5%)
Race		
	White	3956 (74.6%)
	Black or African American	1001 (18.9%)
	Asian	24 (0.5%)
	Other	81 (1.5%)
	Unknown or decline to answer	238 (4.5%)
Ethnicity		
	Hispanic	370 (7.0%)
	Not Hispanic or Latino	4756 (89.7%)
	Unknown or decline to answer	174 (3.3%)
Admission source		
	Home/community	4721 (89.1%)
	Long-term care	280 (5.3%)
	Another hospital	155 (2.9%)
	Other/unknown	144 (2.7%)
Intensive care unit during current admission		1800 (34.0%)
Surgery during current admission		942 (17.8%)
Healthcare exposures, past 3 months		
	Hospitalization	1968 (37.1%)
	Nursing home	221 (4.2%)
	Intensive care	356 (6.7%)
	Surgery	519 (9.8%)
Multi-drug resistant isolate ^1^		636/5117 (12.4%)
Isolate resistance ^2^		
	Aminoglycosides	691/5292 (13.1%)
	Carbapenems	874/4601 (19.0%)
	Extended-spectrum cephalosporin	887/5163 (17.2%)
	Fluoroquinolones	1136/5157 (22.0%)
	Piperacillin	763/5000 (15.3%)
Previous *pseudomonas* infection one year prior to admission		477 (9.0%)
Charlson score ^3^		
	Median (interquartile range)	3 (2–5)
Elixhauser score ^3^		
	Median (interquartile range)	5 (3–7)
Cerebrovascular disease		686 (12.9%)
Chronic pulmonary disease		3648 (68.8%)
Congestive heart failure		1838 (34.7%)
Diabetes without chronic complications		1493 (28.2%)
Diabetes with chronic complications		922 (17.4%)
Dementia		379 (7.2%)
Hemiplegia or paraplegia		434 (8.2%)
Influenza		122 (2.3%)
Malignancy		1288 (24.3%)
Mild liver disease		374 (7.1%)
Myocardial infarction		586 (11.1%)
Peripheral vascular disease		759 (14.3%)
Renal disease		1405 (26.5%)
Tuberculosis		51 (1.0%)

N (%), unless otherwise noted. ^1^ Multi-drug resistant isolate defined as any isolate that tested either intermediate or resistant to at least one antibiotic in at least three of the following categories: aminoglyco-sides, carbapenems, extended-spectrum cephalosporins, fluoroquinolones, piperacil-lin/tazobactam. ^2^ Aminoglycosides (amikacin, gentamicin, tobramycin), carbapenems (imipenem, meropenem, doripenem), extended-spectrum cephalosporins (cefepime, ceftazidime), fluoroquinolones (ciprofloxacin, levofloxacin), and piperacillin/tazobactam. Denominator changes for each antimicrobial category based on whether the isolate was tested for susceptibility to an antibiotic in that category. ^3^ Calculated based on diagnosis during the current hospital admission.

**Table 2:** Treatment heterogeneity in patients with *Pseudomonas aeruginosa* pneumonia.

Treatment Patterns		All Antibiotics (n = 5300)	Any Initial Antibiotic(s), then at least One Anti-Pseudomonal Antibiotic ^1^ (n = 3530/5300, 66.6%)	Any Initial Antibiotic(s), then only Anti-Pseudomonal Antibiotics ^2^ (n = 576/3530, 16.3%)	Anti-Pseudomonal Antibiotic Classes ^3^ (n = 4542/5300, 85.7%)
Unique change patterns with length of therapy, n (%)		4635 (87.5%)	3430 (97.2%)	527 (91.5%)	2294 (50.5%)
Unique change patterns without length of therapy, n (%)		4004 (75.5%)	3049 (86.4%)	339 (58.9%)	1048 (23.1%)
Change in therapy	Number with change, n (%)	4473 (84.4%)	3530 (100%)	576 (100%)	2640 (58.1%)
	Day of change from culture, median (IQR)	1 (0–2)	1 (0–2)	2 (1–3)	3 (1–4)
	Number of changes, median (IQR)	3 (2–5)	3 (2–5)	1 (1–2)	2 (1–3)
	Unique change patterns with length of therapy, n (%)	4331 (96.8%)	3430 (97.2%)	527 (91.5%)	2141 (81.1%)
	Unique change patterns without length of therapy, n (%)	3889 (86.9%)	3049 (86.4%)	339 (58.9%)	1027 (38.9%)
No change in therapy	Number without change, n (%)	827 (15.6%)	-	-	1902 (41.9%)
	Unique non-change patterns with length of therapy, n (%)	304 (36.8%)	-	-	153 (8.0%)
	Unique non-change patterns without length of therapy, n (%)	115 (13.9%)	-	-	21 (1.1%)

IQR = interquartile range. Exposure mapping identified all antibiotics received each day, from three days prior to the culture collection date until discharge, or 30 days from culture for longer hospital stays. ^1^ Subgroup of first column, any initial antibiotic/s, then at least one anti-pseudomonal antibiotic. ^2^ Subgroup of second column, any initial antibiotic/s, then only anti-pseudomonal antibiotics. ^3^ Subgroup of first column, only anti-pseudomonal antibiotic classes assessed, may have received other antibiotics. Anti-pseudomonal antibiotic classes included aminoglycosides (amikacin, gentamicin, tobramycin), carbapenems (imipenem, meropenem, doripenem), extended-spectrum cephalosporins (cefepime, ceftazidime), fluoroquinolones (ciprofloxacin, levofloxacin), piperacillin/tazobactam, polymyxins (colistin, polymyxin B), aztreonam, ceftazidime/avibactam, ceftolozane/tazobactam.

**Table 3:** Clinical outcomes in patients with *Pseudomonas aeruginosa* pneumonia.

Outcomes	Change in Therapy (n = 4473)	No Change in Therapy (n = 827)	*p*-Value	No Change, Monotherapy (n = 591)	No Change, Combination Therapy (n = 236)	*p*-Value
Length of stay (days), admission to discharge, median (IQR)	12 (5–30)	4 (2–12)	<0.0001	5 (2–14)	3 (2–8)	<0.0001
Length of stay (days), culture to discharge, median (IQR)	8 (4–19)	2 (1–6)	<0.0001	3 (1–7)	2 (1–4)	<0.0001
Inpatient mortality, n (%)	765 (17.1%)	85 (10.3%)	<0.0001	35 (5.9%)	50 (21.2%)	<0.0001
Mortality within 30 days of culture ^1^, n (%)	891 (19.9%)	138 (16.7%)	0.03	67 (11.3%)	71 (30.1%)	<0.0001
Readmission within 30 days of discharge, n/n (%)	808/3708 (21.8%)	155/742 (20.9%)	0.58	121/556 (21.8%)	34/186 (18.3%)	0.31
Persistent positive *P. aeruginosa* culture ^2^, n/n (%)	679/1781 (38.1%)	14/153 (9.2%)	<0.0001	12/120 (10.0%)	2/33 (6.1%)	0.73 ^3^
*P. aeruginosa* reinfection within 30 days of discharge, n/n (%)	244/3708 (6.6%)	40/742 (5.4%)	0.23	30/556 (5.4%)	10/186 (5.4%)	0.99

IQR = interquartile range. Percentages compared with Chi-square test, medians compared with Wilcoxon test, unless otherwise indicated. ^1^ Inpatient or outpatient mortality. ^2^ Positive culture for *Pseudomonas aeruginosa* after 7 days of treatment. Denominator only includes patients with follow-up cultures. ^3^ Fisher’s exact test.

## Data Availability

The study data may be made available upon reasonable request and approval by the Department of Veterans Affairs.
